# Intensity-dependent self-induced dual-color laser phase modulation and its effect on terahertz generation

**DOI:** 10.1038/s41598-020-80105-7

**Published:** 2021-01-12

**Authors:** Chen Gong, Iwao Kawayama, Hironaru Murakami, Takahiro Teramoto, Masayoshi Tonouchi

**Affiliations:** 1 Institute of Laser Engineering, Osaka University, Osaka, 565-0871 Japan; 2 Graduate School of Energy Science, Kyoto University, Kyoto, 606-8501 Japan; 3 Institute for Radiation Sciences, Osaka University, Osaka, 565-0871 Japan

**Keywords:** Ultrafast lasers, Terahertz optics, Nonlinear optics

## Abstract

Powerful, broadband terahertz (THz) pulses and its application attract an exponential growth of interests. Dual-color laser filamentation in gases is one of the promising THz sources because of the scalability of the THz energy and wavelength with input parameters. But the additional phase induced by the nonlinearities associated with high intensities cannot be neglected because it may result in modulation of the THz waves. We investigate the influences of the infrared pump energy and air dispersion on the terahertz generation in dual-color laser filament. We observe that optimum dual-color laser relative phase of the THz generation undergoes a linear shift with increasing pump energy due to the intensity-induced refractive index change. This phase shift is verified by the spectral broadening of a two-color laser affected by the same mechanism. The result improves our understanding of the theoretical framework for a higher power THz source.

## Introduction

The terahertz (THz) frequency range lies at the boundary of high-frequency electronics and photonics, which acts as a bridge between the two technologies^1,2^. In recent years, increasing attention has been paid to the nonlinear interactions between strong THz electromagnetic fields and materials, including ultrafast terahertz spintronics^3^, ultrafast thermochemical reaction kinetics^4^, and terahertz biological effects^5^. One of the most powerful table-top sources of THz pulse radiation is generated by a femtosecond (fs) laser-induced filament^6^. This method is usually performed with an intense infrared (IR) pulse from amplified Ti:Sapphire laser system and its second harmonic (*ω* and 2*ω*) which is frequency converted with nonlinear crystals such as type-I β-BaB_2_O_4_ (BBO). In contrast to other ultrafast-femtosecond-laser-based methods using electro-optical crystals^7^, photoconductive switches^8^ or semiconductor wafers^9,10^, laser filament-based method has no damage threshold for the emitter. With 10 fs pulse and its second harmonic focused in air, the corresponding spectral bandwidth can be extended to 100 THz^11^. Such ultrafast THz pulses can achieve very high peak intensities, which are essential to pave the way for further new research and applications of nonlinear interactions.

The THz wave generation process has been explained by a four-wave mixing model based on the third-order nonlinearity *χ*^(3)^ in air^12–14^, or a photocurrent model based on tunneling ionization^15–17^. These correspond to a nonlinear polarization for the THz emission owing to the bound and free electrons respectively. What both models have in common is the dual-wavelength phase matching condition for the effective THz radiation. Such a relative phase can be typically tuned by varying the distance between the nonlinear optical crystal and the focus, that is, adjusting the optical path lengths for *ω* and 2*ω* owing to air dispersion^13,18,19^ or using an in-line phase compensator to introduce an additional relative phase^20^. However, it has been shown that an additional relative phase between the *ω* and 2*ω* is introduced by varying the intensity, thus changing the optimal distance between the BBO and the focal point for THz generation^19,21^. Few in-depth studies on the additional phase associated with the nonlinearity introduced by high intensities have been conducted.

A similar topic is white-light generation during the self-focusing process of ultra-short laser, which is closely related to filamentation^22,23^. At sufficiently high input intensity, an extremely broaden part on the blue side of the emission pulse spectrum appears, which has been explained by intensity-dependent refractive index causing temporal deformation of the laser pulse. Specifically, an additional phase induced by the interaction of high intensity in self-focal region with nonlinear medium and plasma during the filamentation process, i.e., the self-phase modulation (SPM)^24^. Extremely fast and strong local refractive index variation also induces an additional phase to the 2*ω* laser pulse, known as cross-phase modulation (XPM)^25^. Therefore, observing the *ω* and 2*ω* frequency shifts caused by SPM and XPM can be an effective method to provide further understanding of the mechanism of intensity induced THz modulation.

In this study, the evolution of THz radiation from a dual-color laser filament in air is quantitatively investigated by changing the incident IR pulse energy and BBO-to-focal distance. In our experiments, the observed shift in the optimal position of THz radiation implies an intensity dependent additional relative phase. An oscillatory power function is proposed to fit the output THz pulse energy measurement results. The additional phase term is proportional to the IR pulse energy, and the validity of this linear relationship is supported by the measured *ω* and 2*ω* blue shifts due to SPM and XPM. As a complement to the validation of the fit function, the two orthogonal polarization components of the THz pulse are measured, and their ratios are also consistent with the fitting parameters.

## Experimental setup

**Figure 1:**
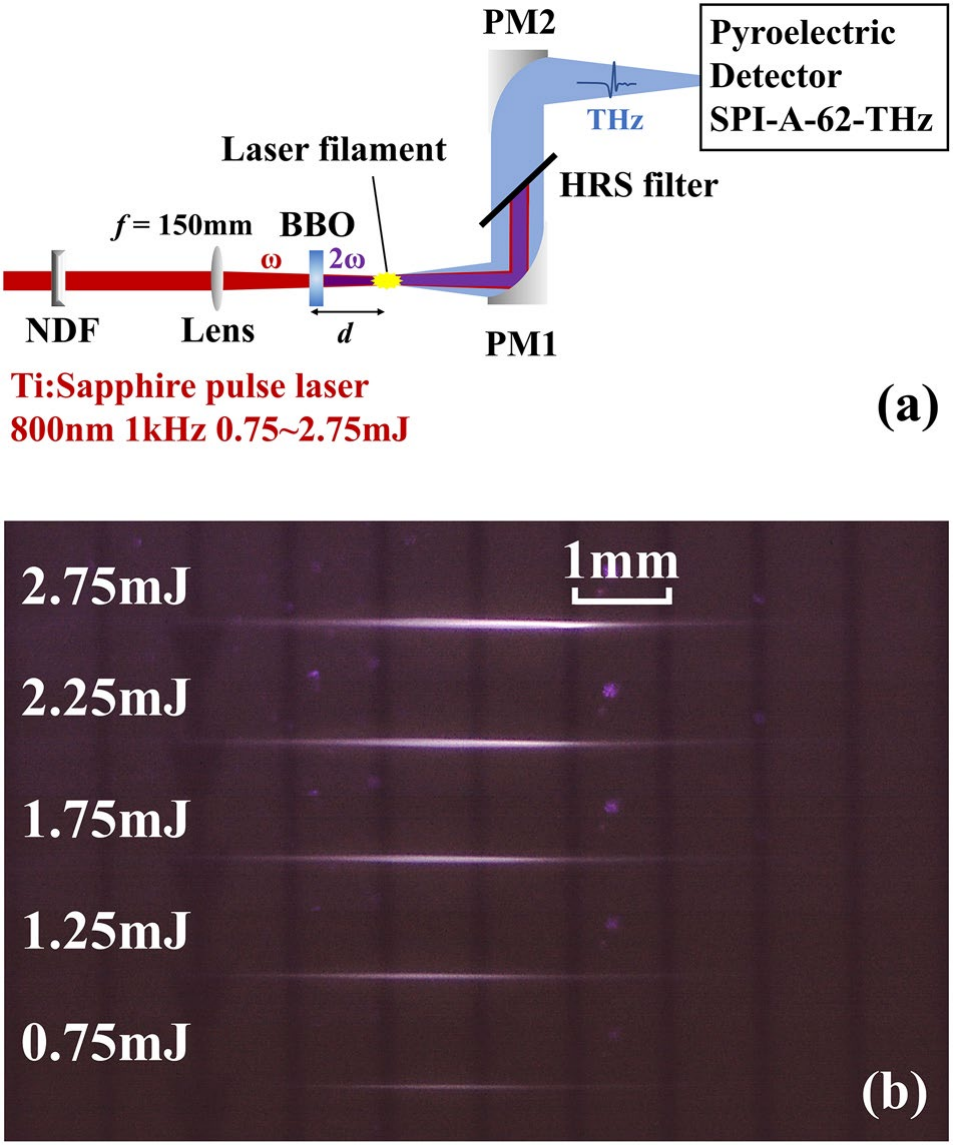
Schematic setup of a dual-color laser filament THz generation system. (**a**) Infrared 800 nm pulse from amplified Ti:Sapphire laser system and its second harmonic (*ω* and 2*ω*) converted with a 0.1 mm type-I β-BaB_2_O_4_ (BBO) co-focused on air at standard atmospheric pressure to form a laser filament as shown in (**b**). The distance *d* between the BBO crystal and the laser filament is adjusted by a linear stage. After the filament, *ω*, 2*ω* and emitted THz pulses are collected by a parabolic mirror (PM1). The ω and 2ω pulses are then filtered by a high-resistance silicon (HRS) wafer, the THz pulses are focused by another parabolic mirror (PM2), and the pulse energy is measured by a pyroelectric detector (SPI-A-62-THz). (**b**) CCD images of filaments taken at corresponding incident pulse energies adjusted by a circular variable neutral density filter (NDF).

All the experiments were conducted at room temperature and standard atmospheric pressure. The experimental setup is shown in Fig. 1a. A Ti:sapphire femtosecond laser amplifier (Spitfire Pro, Spectra Physics) is employed to generate 1 kHz, 100 fs horizontal polarized pulses at a center wavelength of 800 nm. After being focused by a convex lens with a focal length of 150 mm, laser pulses propagate through a 0.1 mm-thick type-I β-barium borate (BBO) crystal to generate second harmonic pulses. BBO crystal is mounted on a one-dimensional linear stage so that the distance *d* between BBO and focus is adjustable in the range of 20–55 mm. Incident plane of BBO is adjusted as perpendicular to incident beam. At the focus a ~ 6 mm long laser filament in air is created by dual-color pulses when the pulse energy is 2.75 mJ. Figure 1b shows a series of photographs of laser filaments versus variate incident pulse energy captured by a charge-coupled-device (CCD) camera. THz radiation from laser filament is collected and collimated by a pair of off-axis parabolic mirrors (PM1 and PM2) after eliminating the pump laser pulses with a high-resistance silicon (HRS) filter. A calibrated pyroelectric detector (SPI-A-62-THz, Gentec-EO), with spectral response from 0.1 to 30 THz, 0.4 nW/Hz^−1/2^ noise equivalent power (NEP) and 33 kV/W responsivity at a chopping frequency of 13 Hz^26^, is used to measure the THz pulse energy with pump pulse energy being adjusted through a circular variable neutral density filter (NDF). To record the frequency shift induced by the laser filament, spectrum emitted directly from laser filament was measured with a spectrometer (USB2000 + , Ocean Optics) when the HRS filter was removed.

## Results and discussion

### THz generation models

In both the four-wave mixing (FWM) and photocurrent (PC) model, the THz electric field *E*_THz_ can be described as functions of incident laser electric field^13–16^:

1 $$E_{{\text{THz,FWM}}} \propto \chi^{\left( 3 \right)} \left| {E_{{{2}\omega }} E_{\omega } E_{\omega } } \right|\cos \left( \theta \right),$$

2 $$E_{{\text{THz,PC}}} \propto f\left( {E_{\omega } } \right)E_{{{2}\omega }} \sin \left( \theta \right),$$

where *E*_*ω*_ and *E*_2*ω*_ are the amplitudes of the fundamental (*ω*) and its second harmonic (2*ω*) electric fields, respectively, and *θ* is the relative phase between these two waves. In addition, *χ*^(3)^ is known as the third-order nonlinear optical susceptibility of the optical medium, and *f*(*E*_*ω*_) is a function describing the dependence of the ionization rate on the incident fundamental electric field *E*_*ω*_, which is expressed variably in different ionization models^8^. Equations (1) and (2) indicate an optimal dual-wavelength phase-matching condition for both models, which affects the effective THz generation when the relative phase *θ* is modulated.

### THz pulse energy and dual-color pulse spectral measurements

**Figure 2:**
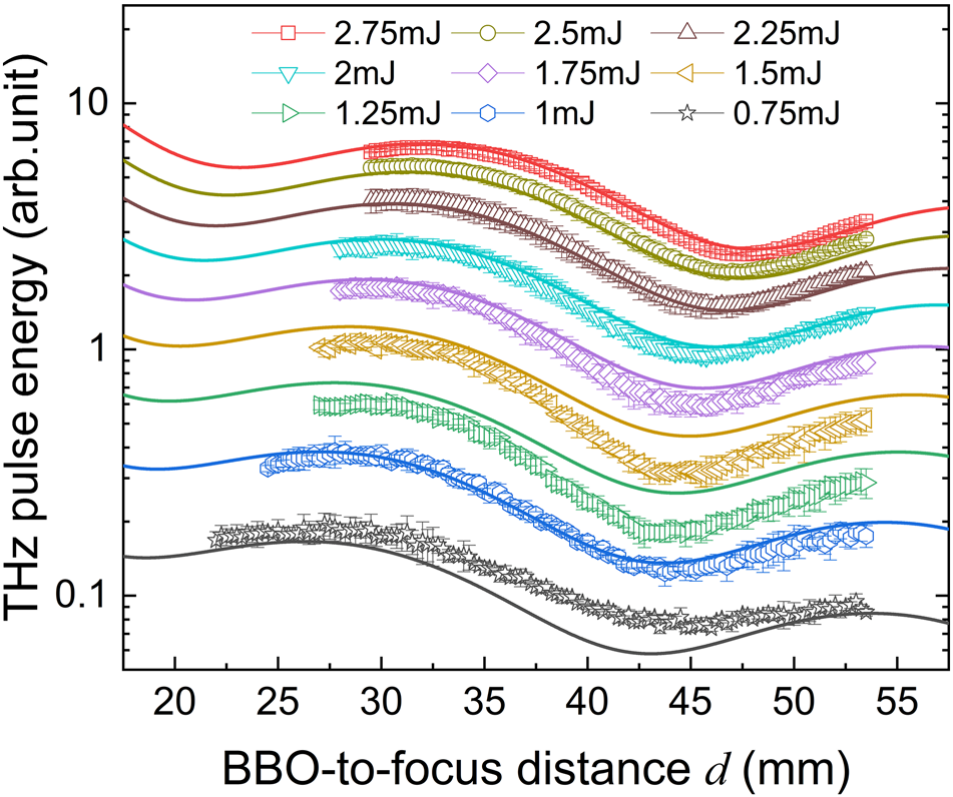
THz radiation evolution by changing the incident IR pulse energy and BBO-to-focal distance. Symbolic points are the THz pulse energy obtained at different type-I β-BaB_2_O_4_ (BBO)-to-focal distance *d* and incident pulse energy *I*_*ω*_ adjusted by an optical attenuator from 0.75 to 2.75 mJ; Solid curves are the fitting results according to an oscillatory power function Eq. (9) described in the “Results and discussion” section. Error bars represent the standard deviation of three measurements.

**Figure 3:**
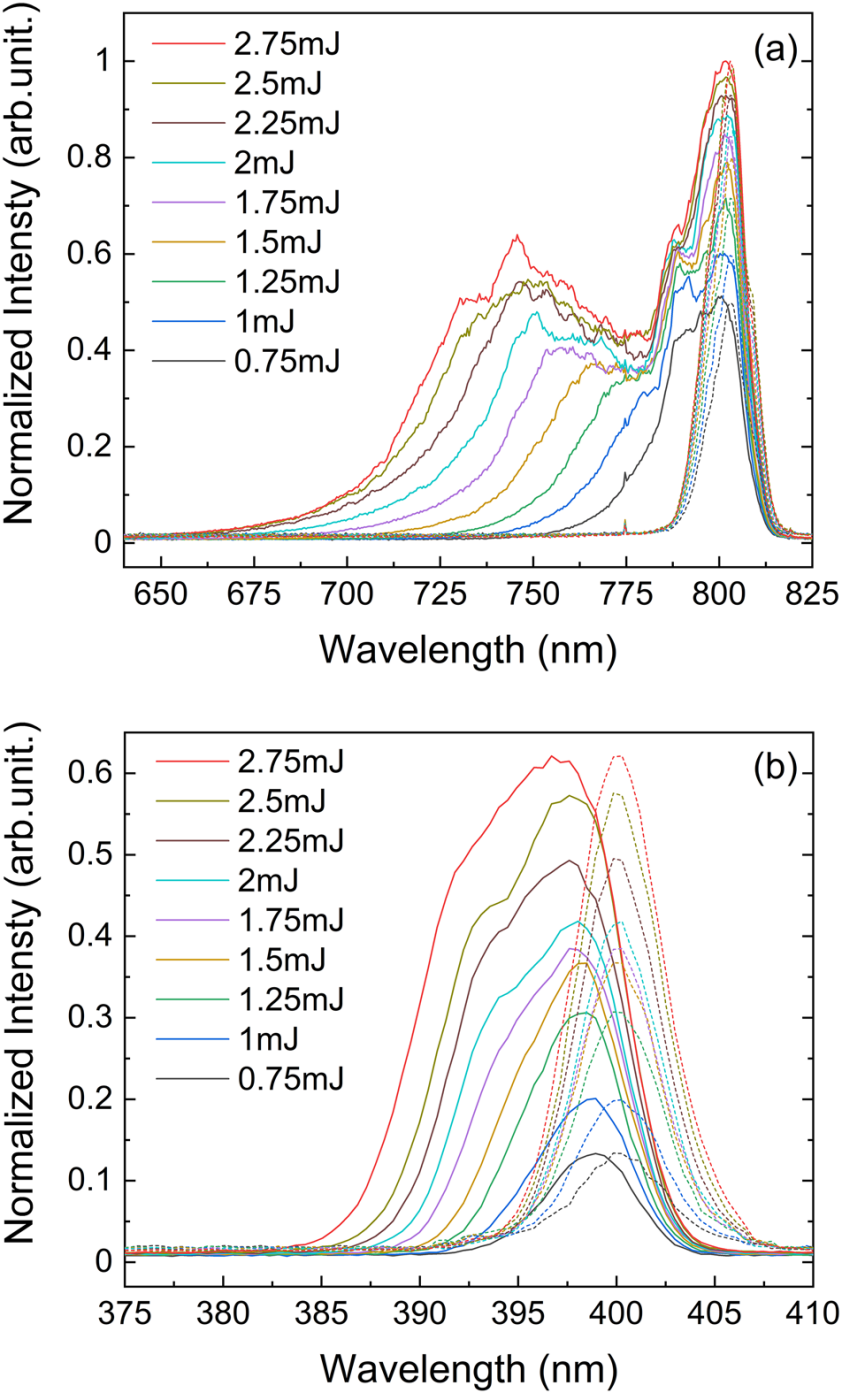
Normalized spectra of fundamental wave *ω* (**a**) and second harmonic wave 2*ω* (**b**). Solid lines are the spectra emitted from a laser filament, and the dashed lines are the spectra measured before the focusing.

Figure 2 illustrates the measured THz pulse energy $I_{{{\text{THz}}}}$ versus the BBO-to-focal distance *d* and incident IR pulse energy $I_{\omega }$ adjusted from 0.75 to 2.75 mJ, which are represented logarithmically for clarity. With an increase in $I_{\omega }$, $I_{{{\text{THz}}}}$ shows an overall exponential growth and exhibits periodic oscillations with *d*. Note that the measured data is the total energy of the THz pulse, not the peak electric field at a particular point in time. According to Eqs. (1) or (2), the oscillations should be represented as sin^2^(*θ*) or cos^2^(*θ*) for $I \propto \left| E \right|^{2}$. This oscillation behavior has been recognized by many studies as the dispersion of air between *ω* and 2*ω*^13,18,19^. It is interesting to note that the extrema position gradually shifted away from the focus with an increase in $I_{\omega }$ The maximum value at a 2.75 mJ pump has a position offset of ~ 5 mm compared to a 0.75 mJ pump, which indicates an intensity-dependent phase shift. To gain insight into the origin of this phase shift, we measured the spectra of the dual-wavelength pulses before and after focusing at different incident pulse energies. Normalized spectra are shown in Fig. 3a,b, in which the central frequency shift and increased broadening on the blue side of both *ω* and 2*ω* were observed as $I_{\omega }$ increased.

### Intensity induced nonlinearity

Frequency broadening introduced by laser filament was first observed in 1970^27^. Since then, the phenomenon has been observed in a variety of media, including liquids^28^ and gases^29,30^, and has been interpreted by Chin et al.^24^ The refractive index of a strong electromagnetic field in air depends not only on the frequency, but also on the spatial and time-dependent intensity *I*(*r*, *t*). In most cases of THz generation from a dual-color laser filament, a transverse Gaussian TEM_00_ mode beam with radially decreasing intensity *I*(*r*) from its center to the edges is often used. During propagation, the wavefront curvature gradually collapses by the spatial intensity-dependent refractive index, which is well known as the self-focusing effect. Also, high intensity (10^12^ ~ 10^14^ W·cm^−2^) at focus leads to tunneling/multiphoton ionization of the gas molecules, resulting in the generation of plasma^31^. The combination of optical Kerr effect, multiphoton absorption and ionization causes the laser pulse to undergo a series of focusing-defocusing cycles in air, maintaining a long-range self-confined propagation and forming a long plasma channel. The macroscopically observed cumulated ionization tracks along the propagation are known as a filament shown in Fig. 1b.

Ultra-short optical pulses also affect the refractive index by means of temporal intensity variation. We consider the temporal electric field as a Gaussian envelope and transform the time variable *ξ* = *t* − *z*/*v*_g_ to a frame comoving with the group velocity *v*_g_ of the laser pulse. For an electromagnetic wave of frequency *ω*_0_, the temporal change in refractive index Δ*n*(*ξ*) affected by the intensity variation is

3 $${\Delta }n\left( \xi \right) = { }n_{2} I\left( \xi \right) - \frac{{\omega_\text{p}^{2} }}{{2n_{0}^{2} \omega_{0}^{2} }}.$$

The first and second terms on the r.h.s. of Eq. (3) account for the nonlinear contribution of the optical Kerr effect and free carriers in plasma respectively. The coefficient of the nonlinear Kerr index *n*_2_ is related to the third order susceptibility by $\chi^{\left( 3 \right)} = 4\varepsilon_{0} cn_{2} n_{0}^{2} /3$^32^, where *ε*_0_ denotes the free space permittivity, *n*_0_ is the linear refractive index of air, *c* is the speed of light in vacuum and *I*(*ξ*) is the temporal Gaussian pulse intensity. The change in the refractive index introduced by the plasma is calculated from an approximation of $\omega_{\text{p}} = \sqrt {e^{2} N_{e} \left( \xi \right)/\varepsilon_{0} m_{e} } \ll \omega_{0}$^24^, where *ω*_p_ is the plasma frequency, *N*_*e*_(*ξ*) is the tunneling/multiphoton ionization generated time-dependent electron density in the air, and *e* and *m*_*e*_ are the electron charge and mass respectively. As the pulse propagates along the *z*-axis, the additional phase introduced by the SPM at time *ξ* is expressed as:

4 $$\varphi_{{{\text{SPM}}}} \left( \xi \right) = - \frac{{\omega_{0} {\Delta }n\left( \xi \right)}}{c}z = \frac{{\omega_{0} }}{c}\left[ {\frac{{\omega_{\text{p}}^{2} }}{{2n_{0}^{2} \omega_{0}^{2} }} - { }n_{2} I\left( \xi \right)} \right]z.$$

Then the pulse frequency change due to SPM is equal to:

5 $$\omega_{{{\text{SPM}}}} \left( \xi \right) = \frac{\partial }{\partial \xi }\varphi_{{{\text{SPM}}}} \left( \xi \right) = \frac{{e^{2} z}}{{2n_{0}^{2} \varepsilon_{0} m_{e} c\omega_{0} }}\frac{{\partial N_{e} \left( \xi \right)}}{\partial \xi } - \frac{{\omega_{0} z}}{c}n_{2} \frac{\partial I\left( \xi \right)}{{\partial \xi }}.$$

In Eq. (5), the electron density *N*_*e*_(*ξ*) is dominated by the ionization rate associated with the incident laser intensity *I*(*ξ*)^33^, which gradually increases with *I*(*ξ*) and reaches a maximum increment at the peak. However, because the electron–ion recombination time is usually much longer than the femtosecond scale of the pulse width^34,35^, *N*_*e*_(*ξ*) should be considered static after the peak. Therefore, the first term on the r.h.s. of Eq. (5) has positive values only, i.e., the frequency shift due to the plasma is blueshift. On the other hand, SPM induced by the Kerr nonlinearity broaden the spectrum of the pulse symmetrically. That is, as indicated by the second term on the r.h.s. of Eq. (5), at the leading edge of the intensity, the frequency change caused by the Kerr effect is negative and appears as a redshift, while at the trailing edge is a blueshift. However, in our experimental results (Fig. 3), we did not observe any redshift in the *ω* laser spectrum after focusing. This indicates that the plasma-induced SPM have counteracted locally the SPM caused by the Kerr effect, or that the Kerr nonlinearity is negligible compared to the effect of the plasma. We thus assume that additional phases introduced by the plasma contribute all of the blueshifts. Set the pulse peak at the time coordinate origin, the SPM induced total phase retardation of a Gaussian pulse with pulse width $\tau_{0}$ can be calculated as follows:

6 $${\Delta }\varphi_{{{\text{SPM}}}} = \mathop \smallint \limits_{{ - \tau_{0} /2}}^{{ + \tau_{0} /2}} \omega_{{{\text{SPM}}}} \left( \xi \right)d\xi \cong \frac{{e^{2} z}}{{2n_{0}^{2} \varepsilon_{0} m_{e} c\omega_{0} }}\mathop \smallint \limits_{{ - \tau_{0} /2}}^{{ + \tau_{0} /2}} dN_{e} \left( \xi \right).$$

In the same way, for a second harmonic pulse with a width of $\tau_{0}^{^{\prime}}$, we have:

7 $${\Delta }\varphi_{{{\text{XPM}}}} = \mathop \smallint \limits_{{ - \tau_{0}^{^{\prime}} /2}}^{{ + \tau_{0}^{^{\prime}} /2}} \omega_{{{\text{XPM}}}} \left( \xi \right)d\xi \cong \frac{{e^{2} z}}{{4n_{0}^{2} \varepsilon_{0} m_{e} c\omega_{0} }}\mathop \smallint \limits_{{ - \tau_{0}^{^{\prime}} /2}}^{{ + \tau_{0}^{^{\prime}} /2}} dN_{e} \left( \xi \right).$$

It can be seen from Eqs. (6) and (7) that the phase shift caused by the filament is related to the integration of $dN_{e} \left( \xi \right)$ over time based on the plane wave hypothesis. Since the electron density variation only occurs near the peak, the total phase shift $\Delta \varphi_{{{\text{SPM}}}}$ of the *ω* pulse through the laser filament can be considered to be proportional to its frequency shift $\Delta {\omega }_{{{\text{SPM}}}}$, i.e., $\Delta \varphi_{{{\text{SPM}}}} \propto \Delta \omega_{{{\text{SPM}}}}$, and similarly $\Delta \varphi_{{{\text{XPM}}}} \propto \Delta (2\omega )_{{{\text{XPM}}}}$. The additional relative phase *θ*_*f*_ of the dual-color laser induced by SPM and XPM can then be written as follows:

8 $$\theta_{f} \propto \Delta \omega_{{{\text{SPM}}}} - \Delta \left( {2\omega } \right)_{{{\text{XPM}}}}.$$

In practice, inhomogeneous intensity distributions near the focal point may lead to invalidation of the plane wave hypothesis. A more general approach is to take the spatial distribution of laser pulses as a three-dimensional function and translate the effect of the lens into a transversely equivalent refractive index, which can be further solved by combining the Kerr self-focusing and plasma self-defocusing effects.

### Fitting function

To investigate the relationship between the dual-wavelength pulses spectral broadening, relative phase and incident light energy, an oscillatory power function as expressed as Eq. (9) below is proposed to describe the THz pulse energy evolution. The relationship between the input IR pulse energy $I_{\omega }$, BBO-to-focal distance *d* and measured THz pulse energy $I_{{{\text{THz}}}}$ was fitted nonlinearly using the standard Levenberg–Marquardt algorithm with a tolerance of 10^−6^ was used. The best fitting result is shown in Fig. 2 as solid curves with the coefficient of determination *R*^2^ = 0.9945, and the fitting parameters are listed in Table 1.

9 $$I_{{{\text{THz}}}} = \frac{{I_{\omega }^{3} }}{d}\left[ {\alpha + \beta {\text{sin}}^{2} \left( {\gamma d - \delta I_{\omega } + \varphi_{0} } \right)} \right],$$

**Table 1:** Fitting parameters of Eq. (9) based on the data in Fig. 2 (SI units).

*α*	*β*	*γ* (fixed)	*δ*	*φ*_0_
5.862 × 10^−6^	5.069 × 10^−6^	0.1211	3.104 × 10^−4^	1.413

### Oscillation term in the function

**Figure 4:**
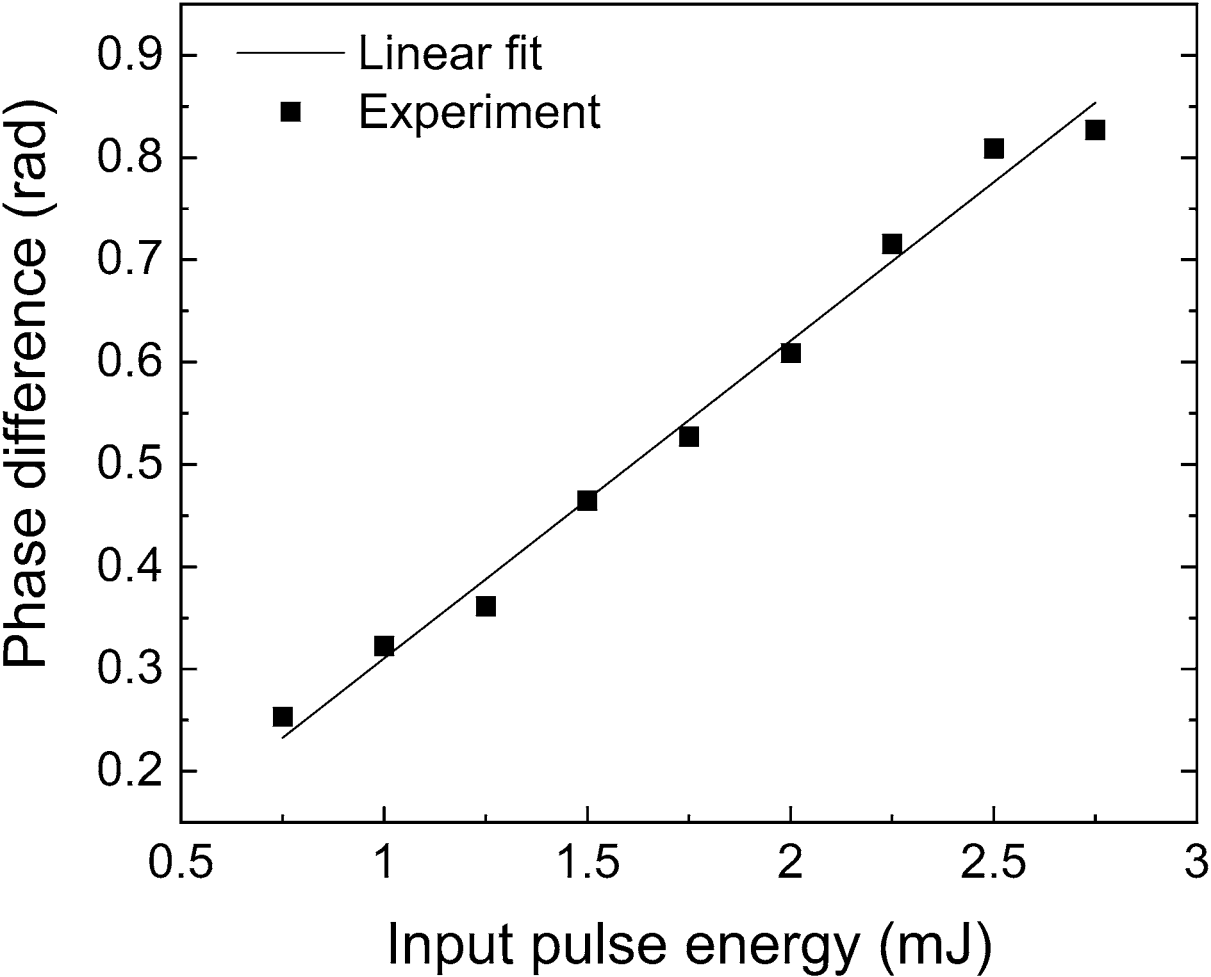
Intensity dependent nonlinearity induced dual-wavelength relative phase evolution. Additional relative phase shift *θ*_*f*_ calculated from Eq. (8) (square) and linear fitting *δI*_*ω*_ from Eq. (9) (solid line) as a function of the incident pulse energy.

We first concentrate on the oscillation-related term in Eq. (9). Because we measured the THz pulse energy instead of the amplitude of the THz electric field, the oscillation term is represented by a square of a sinusoidal function. The *γd* term denotes the dispersion in air calculated using *γ* = 2*ω* (*n*_2*ω*_ − *n*_*ω*_) / *c*, where *n*_*ω*_ and *n*_2*ω*_ are the refractive indices of the 800- and 400-nm waves in air^36^ respectively. The constant term *φ*_0_ represents a series of additional relative phases during the propagation of *ω* and 2*ω* waves that are independent of $I_{\omega }$ and *d*, which can be caused by BBO birefringence^17^, Gouy phase shift^37^, or other factors. The negative phase term *δ* $I_{\omega }$ is understood as the $I_{\omega }$ dependent relative phase caused by the plasma dispersion. To verify it, the phase shifts of *ω* and 2*ω* waves in the laser filament are calculated from Eq. (8), which are obtained by subtracting the frequencies at the 1/*e* peak value of the spectra before and after focusing. The calculated additional relative phases are represented as the squares in Fig. 4, which are fitted by the linear dependent term *δ* $I_{\omega }$ as indicated by the solid line. The measured data are in good agreement with the linear fit, proving that intensity-dependent laser-induced filament affects the relative phase of the dual-color laser field.

### Polarization terms in the function

The constant term *α*, which is the non-zero part of oscillatory curves, has also been observed in previous studies^17,21^. According to Ref.^17^, the polarization of 2*ω* pulse generated from the BBO crystal is not in parallel but rather an angle Θ with incident *ω* pulse. Only the projection component of the *ω* field along the 2*ω* polarization contributes to THz generation. You et al. provided a detailed explanation in their previous study^38^. Owing to the cross action in the high-intensity pump, 2*ω* polarization gradually develops into a rotating elliptical within laser filament, resulting in an elliptically polarized THz emission. Therefore, Eq. (9) can be rewritten as follows:

10 $$I_{{{\text{THz}}}} = \frac{{I_{\omega }^{3} }}{d}\left[ {\alpha {\text{cos}}^{2} \left( {\gamma d - \delta I_{\omega } + \varphi_{0} } \right) + \left( {\alpha + \beta } \right){\text{sin}}^{2} \left( {\gamma d - \delta I_{\omega } + \varphi_{0} } \right)} \right].$$

**Figure 5:**
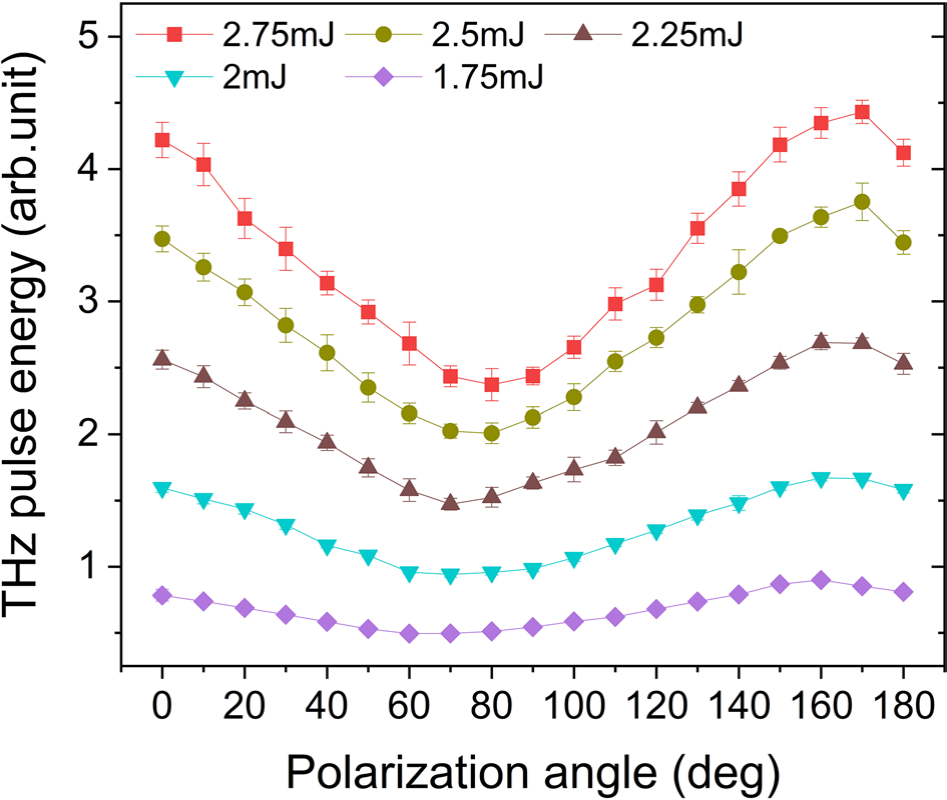
THz pulse energy versus polarization angle and input pulse energy. Polarized THz pulse energy is measured at the corresponding peak position in Fig. 2. The polarization angle is adjusted by a wire grid polarizer in front of the pyroelectric detector. Error bars represent standard deviation of five measurements.

Equation (10) can be recognized as a superposition of two orthogonal polarization components. To verify it, we added a wire grid polarizer in front of the pyroelectric detector to measure the THz pulse energy in different polarization directions at the corresponding peak positions in Fig. 2. The measured results are shown in Fig. 5. The ratio of the two polarization intensity extremes is consistent with Eq. (10), which further verifies the accuracy of the fitting.

### Discussion on the cubic term

In Eq. (10), the emitted THz pulse energy $I_{{{\text{THz}}}}$ is proportional to $I_{\omega }^{3}$, and inversely proportional to *d*. Because the second-harmonic generation efficiency is a function of squared incident intensity: $I_{2\omega } \propto I_{\omega }^{2}$^32^, the 2*ω* pulse intensity $I_{2\omega }$ can be approximately considered as $I_{2\omega } \left( d \right) \propto I_{\omega }^{2} /d$. The agreement between the cubic function and experimental results seems to imply that THz radiation should mainly contribute to the photocurrent rather than to the four-wave mixing, because the dependence of $I_{{{\text{THz}}}}$ on $I_{\omega }$ does not obey the power laws predicted by Eq. (1). However, in the case of short focusing, the spatio-temporal simulation becomes extremely demanding because it involves large different scales of the incident pulse intensity distribution *I*(*r*, *t*). As far as the experimental results involved in the preset paper are concerned, we cannot conclude on which mechanism contributes more to THz generation.

## Conclusion and outlook

In summary, by measure the THz pulse energy evolution we observed that the relative phase of the incident dual-wavelength pulses, an important factor in the modulation of THz generation, is modulated not only by the additional dispersion by air, but also by the temporal refractive index changes due to the self-induced intensity dependent nonlinearity. An oscillatory exponential function is proposed to fit the observed THz pulse energy evolution, by which the relative phase causing the THz modulation is found to be proportional to the incident pump pulse energy. And the validity of this curve fitting is also confirmed by the SPM and XPM modulated dual-color filament spectra and THz polarization orthogonal component ratio. Although the phase modulation at short focal lengths is not as pronounced as in the case of long laser filaments, the observed nonlinear effects introduced by the high-intensity light field are still not negligible and provide further insights into the mechanism of formation and evolution of laser filaments in air. However, this article cannot account for the scaling law of THz energy versus incident IR energy, i.e., the cubic term in the fit function, which requires accurate spatiotemporal modeling of filaments in this particular experimental parameter regime. This investigation will be helpful to those working on the principles of a laser filament THz generation mechanism, and will also be useful for building intense wideband THz sources and systems for further application experiments.

## Data Availability

The data that support the findings of this study are available from the corresponding author upon reasonable request.
